# Eating psychopathology and psychosocial impairment in eating disordered individuals - a Singapore study

**DOI:** 10.1186/2050-2974-1-S1-O68

**Published:** 2013-11-14

**Authors:** Kah Wee Ng, Angeline Kuek, Huei Yen Lee

**Affiliations:** 1 Department of Psychiatry, Singapore General Hospital, Singapore

## 

Research has shown that eating disorders have a serious and detrimental impact on an individual's physical and mental well-being. In various studies conducted in the West, it was seen that eating disorders have a negative impact on psychosocial functioning and that individuals with eating disorders report a lower quality of life. It is interesting to note that previous studies have shown that south-east Asian individuals exhibit lower levels of body satisfaction, greater eating disorder psychopathology, and more concern about their weight compared to their Western counterparts. However, compared to Western individuals, the level of eating psychopathology and psychosocial impairment in Asian individuals is still relatively unknown. Knowledge in these domains may improve treatment and elucidate the impact eating disorders have on Asian individuals. The objectives of this study are:

a) To determine the level of eating psychopathology and psychosocial impairment in individuals with eating disorders in Singapore and;

b) To determine if there are any differences in levels of eating psychopathology and psychosocial impairment between the different eating disorders classifications.

This abstract was presented in the **Understanding and Treating Eating Pathology** stream of the 2013 ANZAED Conference.

